# The challenges of military medical education and training for physicians and nurses in the Nordic countries - an interview study

**DOI:** 10.1186/s13049-017-0376-y

**Published:** 2017-04-11

**Authors:** Linda Sonesson, Kenneth Boffard, Lars Lundberg, Martin Rydmark, Klas Karlgren

**Affiliations:** 1 Department of Learning, Informatics, Management and Ethics (LIME), Karolinska institutet, Tomtebodavägen 18A, 171 77 Stockholm, Sweden; 2 Department of Surgery, Milpark Hospital Academic Trauma Centre, University of the Witwatersrand, Johannesburg, South Africa, Guild Road, Parktown ZA, Johannesburg, 2193 South Africa; 3 Institute of Clinical Sciences, Department of Surgery, Sahlgrenska Academy, University of Gothenburg, 413 45 Gothenburg, Sweden; 4 Mednet, Institute of Biomedicine, Sahlgrenska Academy, University of Gothenburg, Medicinaregatan 7B, 405 30 Gothenburg, Sweden; 5 Swedish Armed Forces Centre for Defence Medicine, Götaälvsgatan 20, 426 76 Västra Frölunda, Sweden

**Keywords:** E-learning, Military medicine, Medical education, Web based learning, Blended learning, Distance learning, Technology-enhanced learning

## Abstract

**Background:**

Development and use of e-learning has not taken place to the same extent in military medicine in the Nordic countries. The aim was to explore the similarities and differences in education and training in military medicine for health professionals in the Nordic countries, and more specifically to identify the specific challenges regarding education and training of military medicine, and how e-learning is used at present and the opportunities for the future.

**Methods:**

Key educators within military medicine in the Nordic countries, as approved by the respective Surgeons General, were interviewed and the interviews were analyzed using content analysis.

**Results:**

The data showed that all Nordic countries cooperate in the field of military medical training to some extent. The models of recruitment and employment of health professionals differed as well as the degree of political support. These differences affected the ability for health professionals to gain actual experience from the military environment. To improve the quality of medical education and training, attempts were made to recruit physicians. The recruitment of physicians was considered a challenge which had resulted in disruptions of courses, training programs and maintenance of accreditation. The Nordic countries were described as having commonalities in the military medical systems and common needs for international collaboration within training, but differing in the range of education and training. Gaps were identified in methods for transferring outcomes from education into practice, as well as regarding evaluation and feedback of outcomes to military medicine. The educational tradition was described as oriented towards practical skills training without requirements on pedagogical education of educators. The results confirmed previous studies showing that e-learning was underutilized. Contextual understanding and experience of healthcare were seen as crucial factors for successful e-learning development.

**Conclusions:**

Extended Nordic cooperation on military medical education and training are needed because of the limited volumes of advanced trauma cases. A key issue to the success of e-learning and blended learning is combining educational competence with contextual understanding into a strategy, of how to use digital educational methods.

## Background

Modern war and its competing mission priorities of asymmetrical combat, peacekeeping, humanitarian missions and beneficiary care places great demands on the requirements for military medical education and training [1, 2]. In the Nordic countries, the number of full-time military health care providers is limited and extensive use is made of physicians and medical workers whose main area of work is within the civilian sector. Military medical units in the Nordic countries have an established collaboration within the military medical training domain, for example through the *Nordic Defence Cooperation* [3]. In contrast, collaboration seems to be more limited in education, like for instance by running Nordic joint courses, even though the countries in general have similar civilian and military healthcare models.

Sustainable education and training can take place in military medical facilities, civilian institutions, or in a combination of the two [4–6]. New demands not only create problems, but also grant opportunities to improve, such as suggested in a recent Swedish Government decision to improve military and civilian interchange in trauma [2]. Collaboration between military medical units in both education and training and in a civilian and military setting might be one of the key ways to meet the new demands [2, 4], especially since the Nordic countries do not have the volumes of trauma patients or developed systems of trauma care to uphold adequate advanced trauma education and training. Working as a health professional in military medicine demands direct patient care during extreme environments, or experiences of medical work in military environments [1, 4, 7].

When the opportunities for experience within the military environment are scarce, education and training strategy supported by digital technologies will be essential for maintaining skills [1, 4–6]. Combining educational practices like face-to-face lectures with the use of digital technologies is known as blended learning. In blended learning environments, face-to-face lectures are mixed with distance learning by using digital technologies such as, web-based learning systems and digital mannequins as support for learning [4, 8–10]. An optimal education and training environment will benefit from all the strategies mentioned, used in combination with each other, or separately [4, 9, 11].

Blended learning supports the use of interactive multimedia and collaboration in education and training and has become the tool of choice in education [12–14]. One form of online courses aimed at unlimited participation and open access are the Massive Open Online Courses (MOOC) [6, 9, 15, 16]. However the use in military medicine until now has been limited [6, 9].

There are some examples of e-learning developed specifically for military medicine, such as the NATO Centre of Excellence for Military Medicine which has developed e-learning courses for distance learning in military medicine, and the International Committee of the Red Cross, which has developed e-learning for healthcare professionals working in armed conflicts and other emergencies [17, 18]. These courses are also mainly distance courses offering interviews with experts in the field and various learning activities. The website Trauma.org is an independent, non-profit organization which provides practice scenarios called *“trauma moulage scenarios”* online [19]. There is however a lack of research on the development, use and outcomes of e-learning for military medicine and in blended learning contexts [4, 6, 9, 20].

### Aim

The aim was to explore the similarities and differences in education and training in military medicine for health professionals in the Nordic countries of Denmark, Finland, Norway and Sweden, and more specifically to identify the specific challenges regarding education and training of military medicine, and how e-learning is used at present and the opportunities for the future.

The research questions were:

1. How is education in military medicine organized in the Nordic countries?
2. What are the specific educational and pedagogical challenges in military medicine in the Nordic countries?
3. How is technology-enhanced learning being used in military medicine in the Nordic countries today and what are the plans for its use in the future?

## Methods

### Design and setting of the study

Semi-structured interviews were carried out with key educators within the military medical education systems in the Nordic countries. The interviews took place within each country.

Military clearance by the Surgeon General of each respective nation was obtained and collectively identified 11 respondents and gave the permission for interviews. The interviewees were all serving as officers at the rank of major or above in the armed forces of Denmark, Finland, Norway and Sweden. Nine were male, two were female. The interviewees held key positions as directors of studies and educators in the domain. There were four physicians, three registered nurses and four non-medical personnel. All of the interviewees were also involved in development of educational practice and policy.

Information about the study was given both verbally and through an information letter. The identities of the participants in this study have been anonymized by using a numbered code for each informant, for example Personal Interview number 11 (P11).

#### Semi-structured interviews

A semi-structured interview provides the researcher with guidance through a number of topics but allows diverting as the semi-structured interview is open, allowing new ideas to be brought up during the interview as a result of what the interviewee says [21]. A framework consisting of a number of interview questions was prepared and was used to focus on topics to be covered but allowing the interviewer to ask questions in different ways for different participants and allowing the interviewer to follow topical trajectories in the conversation that strayed from the guide when this was appropriate. As the guide did not constrain the interview to a particular format questions could be tailored to the interviewee and the interview situation. Their responses were documented, and the dialogue allowed for them to develop the topics of discussion. Therefore, subsequent questions were targeted towards areas that could be explored more fully. The guide included the following questions (Table 1).

**Table 1:** The semi-structured interview guide

1. How has preparation for extreme situations been handled in the area of military medicine? 2. On which kinds of views on knowledge and education have these educational program been based on? 3. Do course directors, teachers, instructors or similar have formal training in education? 4. Do you use medical simulation, if so what kind? 5. Do you use virtual environments/serious games for training or education in military medicine? 6. What kind of information technology or other supportive technologies have been used in (e.g., learning management platforms)? 7. How common is it to use IT or other technologies to support the course participants’ learning processes in education and training? 8. How do you evaluate and measure outcomes of education and training? 9. Do you know if the participants’ skills match the intended learning outcomes of the courses? 10. How does the military medical education system use or manage skills from education and training? 11. Do you collaborate with other countries in education and training for health professionals, is so which countries and networks and in which purpose? 12. How can IT or other technologies support education and training for health professionals?	

### Data analysis

**Fig. 1:**
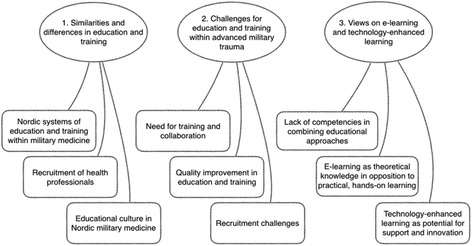
Thematic map showing categories related to each research question

A content analysis was used to identify and analyze categories describing the educators’ descriptions of education and training in military medicine, its specific challenges and views on the use of e-learning. Research using content analysis focuses on a systematic procedure to extract the main content of a text [21–23]. The interviews were read individually and then discussed in the research group. Ideas of interest for the purpose of the study were marked in the text, and ideas about what was in the data were written down. Meaning units were recognized, condensed, and abstracted into categories. The categories were discussed and reviewed in relation to the coded groups of text and to the entire dataset. The specifics of each category were refined and were discussed until agreement was reached. The analysis involved moving back and forth between the dataset, the coded text, and the ongoing analysis of the data. During the entire analysis process, discussions among the researchers were continually held to ensure rigor toward data and contribute to coherence of the findings. The outcome of the analysis was grouped into six categories answering the three research questions (Fig. 1).

## Results

This section is organized based on the three research questions. The categories discovered by the content analysis will be presented with the corresponding research question.

### Similarities and differences in education and training

Three categories regarding the first research question about similarities and differences in military medical education were discerned: *The Nordic systems of education and training within military medicine, Recruitment of health professionals,* and *Educational culture in Nordic military medicine*. Each theme is presented below with quotations to illustrate the findings.

#### The Nordic systems of education and training within military medicine

The interviews revealed that the models of recruitment and employment of military health professionals differed between nations as well as political support and resources. All the Nordic countries cooperated in the military medical domain related to training for battlefield. Denmark and Sweden were described as having a close collaboration in naval medicine as well. Norway and Sweden have similar military medical educational systems, centralized to a centre or a school. In Denmark and Finland, military medical education was located to several different training platforms. The respondents indicated that Norway and Sweden have a close collaboration in military medical education and training for health professionals. The knowledge of the physicians is used to improve quality of the medical content within education and training.

#### Recruitment of health professionals

The recruitment and employment of physicians was considered to be of great importance for education in military medicine. The focus in Denmark and Norway was mainly the recruitment of surgeons, but Norway also focused on developing trauma teams. The interviewees reported that there were established supportive structures for the recruitment and training of health professionals in military medicine such as political support, resources and close collaboration with universities and hospitals with trauma centers. The supportive structures worked well but there were challenges (see the section on challenges below). Denmark excelled by having developed a recruitment system where physicians were offered specialization in traumatology during their basic medical education and various forms of contracts in combination with permanent positions after graduation. This often resulted in a close relationship with the physicians and their relatives, starting during the university years and continuing during the years of service in the armed forces.

#### Educational culture in Nordic military medicine

All respondents in the Nordic countries described that military medical education was dominated by an educational culture marked by its military tradition including short theoretical briefings and practical training sessions carried out during a distinct and intense course time. The military medical units did not have any requirements of pedagogical education for academic representatives, course managers, and teachers or similar. However, educational experience was nevertheless described as desirable. Interviewees in Denmark were committed to maintaining traditional education with a focus on theoretical briefings, practical training and intensive course periods within the framework of educational programs for health professionals. The importance of being able to control the level of knowledge and content to educate the target group properly, was reported to be one of the causes. In Norway, the military medical education was characterized by having a particular focus on teamwork, the importance of a educational development within the organization was stressed, in accordance with the current developments in civilian higher education. Swedish military medical education differed markedly by profiling academic medical skills combined with the culture of military training but did not define the difference between education and training, and did not use the academic connections within external academic environments:

*“…We make no distinction between education and training… it is a mistake and we are mainly focusing on training… The balance between theory and practice is deficient and the education system in general lacks academic connections…”* [P01]

Even though the military medical education in Sweden was characterized by several active PhDs employed, the military medical educational system did not make use of these academic resources to strengthen the development of the field and improve courses. The Swedish interviewees argued that the balance between theory, practice and research needed to be further optimized, in order to contribute to development of actual and sustainable military medical courses for health professionals.

The interviewees in Norway considered teamwork to be central in trauma management, especially in the operational environment where trauma management is conceived of being a collaborative task making use of competences of all members of a team. After completing advanced trauma education the team returned with knowledge to their respective hospitals but supportive structures for implementation of the knowledge at the hospitals seemed to be unclear. Interviewees in Sweden and Norway highlighted the need of developing team-focused military medical courses within a hospital context for the target group because teamwork is an implemented model of managing emergency care.

However Sweden was distinguished by offering a wide range of general courses for military health professionals in military medicine, in contrast to Denmark, Norway and Finland which offered few general courses. Courses or lectures for health professionals were offered prior to an international deployment or a major training. Health professionals needed to apply for access to courses and the application also needed to be approved by platoon commanders or representatives. In addition Sweden and Norway offered opportunities for civilian health professionals to register for advanced military trauma courses.

### Challenges for education and training within advanced military trauma

Three categories are presented in relation to the second research question about challenges for education and training within advanced military trauma. The categories were: *Need for training and collaboration, Quality improvement* and *Recruitment of health professionals*. Each category is presented below with quotations to illustrate the findings.

#### Need for training and collaboration

All interviewees emphasized the need to develop skills in advanced military traumatology through national and international collaboration and claimed that the need had increased considerably during the last 10 years. According to the interviewees the need was related to the development of trauma as a subspecialty and the reduction in the number of extensive open surgical procedures in the communities. Denmark and Norway had national, as well as international experiences and competencies in advanced traumatology which had contributed to the development of national trauma structures and several trauma centres, in contrast to Sweden which only has one national trauma centre and a lack of political support, and therefore lacked national supportive structures for trauma:

“…*it is a political issue and we are usually placed in the war zones of more prominent position than the Swedes… But the losses are even more substantial, we lost 36 soldiers during the years in Afghanistan…*” [P 11]

In addition to the extended need for education and training in advanced civilian and military trauma all interviewees emphasized the need for improved collaboration between the Nordic countries on education and training for the target group health professionals. The Nordic countries are small and have limited volumes of trauma cases themselves. For this reason international collaboration e.g. with NATO in education and training and the Nordic countries in training was highlighted as important. Through joint education and training, the Nordic countries were able to offer and accomplish specific courses, exchange experiences and knowledge as well as evaluating outcomes from joint education and training. International collaboration within military medical education for the target group health professionals was considered to be established through networks related to external courses (such as Battlefield Advanced Trauma Life Support (BATLS™), Advanced Trauma Life Support (ATLS®), Military Operation Surgical Training (MOST™) and Definitive Surgical Trauma Care (DSTC™)). Established international collaboration and a trauma exchange programmes within advanced traumatology were mainly present in Norway and Sweden. Only Norway and Sweden invest in research to improve education in advanced trauma for health professionals, however its extent is sometimes limited.

Interviewees in Sweden also claimed a need for development of courses which could complement the military medical education by offering training throughout the continuum of care, as well as interaction with the civilian health care. The reason for this need was reported to be a lack of capacity and knowledge within civilian personnel of how to handle high-energy military trauma and mass casualties.

#### Quality improvement in education and training

According to interviewees in Denmark, Finland and Sweden another challenge and an identified need was the lack of feedback to the military medical education systems when it comes to analysis and evaluation of courses. It has not been possible to analyse outcomes from education and training, since the information has been considered and classified as secret. The analysis of courses and training was considered important to be able to obtain knowledge if the content of the courses met the actual needs and maintained health professionals’ skills:

*“…Reports have previously been classified as secret. It is a dilemma for us because we get no feedback about practical outcomes from education and training in the battlefield. No organized feedback to the education system… How do we then know if we are educating and training the right skills?…”* [P05]

The interviewees from Norway highlighted digital support as an area of interest in future evaluation of courses, with a focus on the possibility of measurement of outcomes from education and training.

#### Recruitment challenges

Appointing physicians for permanent positions within military medicine was brought up as a challenge in the interviews. The challenges related to that senior professionals needed a certain proximity to their regular academic environments, as well as a long-term development of individual skills in military medicine, including deployment on international missions. The majority of positions for physicians were located in smaller communities without an academic environment. In Sweden recruitment of physicians, political support and availability of resources were seen as the greatest challenges and affected the opportunities and the possibilities to accomplish education and training.

### Views on e-learning and technology-enhanced learning

Three categories emerged in relation to the third research question about the use of e-learning in military medicine in the Nordic countries: *Lack of competencies in combining educational approaches*, *E-learning as theoretical knowledge in opposition to practical, hands-on learning,* and *Technology-enhanced learning as potential for support and innovation*. These are presented below with representative quotations to illustrate the findings.

#### Lack of competencies in combining educational approaches

The interviewees from Denmark, Finland and Norway indicated that they lacked a general overview of the development of educational approaches and applications of digital support to achieve flexible learning for professions and units. Nor did they had any established dialogue or collaboration with civilian medical education and training environments.

The costs of practical training for physicians and nurses were highlighted as great challenges by the interviewees in the Nordic countries. The possibilities of using e-learning with the purpose of allocating time to use for team training was highlighted as important by the interviewees in Finland, Norway and Sweden. The Interviewees from Finland, Norway and Sweden shared the experiences in the past of using web-based educational techniques such as learning management systems as support for distance learning, where courses were running on internet without face-to-face lectures, but without any success due to the lack of pedagogical competencies about e-learning. Common and shared challenges such as insufficient Internet access for military units, difficulties to examine course participants and the management of courses in a web-based environment were cited as contributing factors to the pitfalls of e-learning in the Nordic military context.

In contrast to the interviewees in Finland, Norway and Sweden the Danish interviewees stated that they had decided not to develop digital support for education and training, as the focus was on traditional face-to-face education:

*“…No, there is no need for it (e-learning)… They* (the *course participants*) *will enter and put themselves in the classroom… They receive information and theory… they participate in discussion groups… And learning takes place in practical assignments and discussions…”* [P08]

According to the Norwegian interviewees, understanding of the context and having experience of healthcare were the crucial keys for success of development within the field of digital support for education and training. Contextual understanding and knowledge within healthcare was seen as important, in order to be able to identify and meet the needs for e-learning in education and teaching. In Sweden, military medicine in cooperation with other parts of the armed forces, focused on digital support and web-based learning platforms supporting flexible learning for the military units and staff. Professional development of teachers was integrated with the development of web-based courses into the environment of a web-based learning management system. Collaborations with civilian medical universities, and international networks were considered as an important part of the development in the area.

#### E-learning as theoretical knowledge in opposition to practical, hands-on learning

One category emerging from the data describes a view of e-learning as something in contrast to and even in conflict with practical, hands-on learning. E-learning was instead viewed as something suitable only for learning theoretical knowledge and not having a place in supporting, extending or analyzing practical exercises. Collaboration and deepening learners’ understanding of the hands-on work was not conceived as taking place in connection with the use of e-learning.

“*… because I believe learning takes place in a physical space between people with different backgrounds and education. So there will be no pushing for e-learning and as long as we are in this practical world there is already much theory but not enough practice. Theoretical parts may be e-learning…*” (P07)

E-learning was according to this category indicated as something detached from the real, practical world where the actual learning was considered to take place and the role of e-learning was limited to providing theoretical background knowledge and of minor importance in military medical education and training.

#### Technology-enhanced learning as potential for support and innovation

Experiences of using digital technologies for education or training were shared by all the Nordic countries. This category describes how the use of technology was seen as a potential support for education and a possibility to innovate training. Besides web-based learning, several other kinds of technologies were suggested as possibilities to enhance learning. This category also emphasized different kinds of learning and not only of theoretical knowledge.

Most commonly, digital support for training and practice were found in the field of medical simulation with digitalized manikins. Technology was also described as having potential for developing simulation scenarios and for electronic cases reflecting patients’ flow through chains of care.

“*…There’s a great potential for developing e.g. sequences of events for simulating e.g. monitoring or x-ray images. So that we can use IT and technology during scenarios. For instance if you have a serious situation at the ICU then you could use electronic cases which support the entire chain of care. To reflect the individual patient’s flow from prehospital to hospital etc…*” (P02)

Moreover, in Finland, mobile digital sensors had been used in combat simulations to display vital parameters of injured patients and to provide instructions and information about time to medics. All countries had experiences of using digital games for learning purposes, although not in games focusing on military medical functions. The interviewees argued for the importance of medical simulations within education and training to be able to prepare and support health professionals through exposure to the extreme environments and characteristic for military medicine. The extreme military environments are difficult to visualize and medical simulation with digital manikins and digital games were considered to have the potential to contribute to be a support. Norway and Sweden were described as addressing the need for improvement of digital manikins and other simulators due to their limitations to authentically simulate aspects of reality. For example the problems of simulating blood circulation and coagulation were viewed as important for training the treatment of ballistic wounds.

## Discussion

### Similarities and differences in education and training

#### Nordic systems of education and training within military medicine

Military medicine as well as civilian health care in the Nordic countries have several commonalities and have established international collaboration within training its personnel. By developing educational collaboration, the need of sharing resources by exchanging course directors, educators/instructors and course participants could be met. Military medicine in the Nordic countries, and in Sweden in particular, were described as having limited experience of external collaboration with civilian stakeholders in medical education and research. To be able to make use of complementary competencies and strengthen the uniqueness of the military medicine field, an extended external collaboration is crucial [1–3]. External collaboration with stakeholders in healthcare such as academic environments and hospitals with trauma profiles might contribute to strengthen the research and development of the field of military medicine as well as make use of academic evidence-based methods in education and training like evaluations and quality improved content of courses.

#### Recruitment of health professionals

The models of recruitment and employment of military medical professionals as well as the degree of political support and resources differed between nations. According to the interviewees, political support and recruitment affected the possibilities for physicians and nurses to acquire experience of the military medical environment in battlefields. Recruitment and employment of physicians was considered to be of great importance for the military medical education. Some countries experienced difficulty in recruiting surgeons. Physicians’ medical knowledge was also used to determine and improve the quality of the medical content within education and training. Challenges in deployment of educators with a background in medical education can affect quality of content in education and training and can leads to disruption of courses and programs and accreditation standards may not always be met [1, 4].

#### Educational culture in military medicine

All respondents commented that military medical education was dominated by traditional military education and training philosophies, with the focus on solving tasks during limited time and extreme environments. The traditional military educational culture affects education and training in military medicine. The courses were often consisted in short theoretical briefings, practical training sessions during a distinct and intense course time, with limited room for reflection and critical thinking.

The educational competence is one of the key issues to success in e-learning. Combining educational approaches into a sustainable strategy for education and training will demand a shift from the traditional military educational approach and make more use of digital technologies to supporting learning in order to achieve blended learning [1–3, 5, 6]. Current approaches to education and training including the use of e-learning in a blended learning context, typically involve shifting focus from the educator-centered dialogue to a participant-centered dialogue and makes use of digital support as support for learning processes and methods for collaboration [2, 7–9].

Shifting the focus might also raise the question of what learning, as a concept, stands for within education. Health professionals in a military medical setting are well educated and academically specialized but often lack the time for education and training, and may have another relationship to learning compared to the traditional military education culture. Recent developments in e-learning and blended learning within medical education strive to identify learners’ needs for sustainable learning and education [3, 7, 8]. The courses supported by e-learning match those needs in relation to the organizational need for competencies. A shift in military medical education creates great challenges of both value-based questions what learning is and how to deal with a mixed culture consisting of health professionals and military needs [2, 5].

Another crucial key to achieve the goal of blended learning in military education and training was educational knowledge in combination with a contextual understanding. Contextual understanding and knowledge within healthcare was seen as important to be able to identify the needs and what kind of digital support in education and teaching. There were no requirements in any of the countries for pedagogical education of educators, only educational experience was required. The military medical units lacked overviews of educational developments in related fields; nor did they have any collaboration or connections with external educational stakeholders. These shortcomings can affect both content in military medical education and training, and its distribution. The lack of pedagogical education for the educators and collaboration with external educational stakeholders also contributes to a vulnerable military medical unit and the risk of disruption of programs or courses [1, 2, 6].

### Challenges for education and training within advanced military trauma

Interviewees in military medical education and training identified lack of exposure to the injury panorama and conditions of military medicine for health professionals. Allocating time for health professionals to participate in courses was seen as a great challenge by the interviewees and therefore it is of great importance to establish collaborations with civilian trauma care [10]. Education and training for health professionals in military medicine demands an exposure to direct patient care during extreme environments or experiences of medical work in military environments.

When opportunities of experiences in military environment, or to maintain skills, cannot be provided, a strategy of blended learning for education and training will be essential [1, 4, 6]. Some military medical units have collaborations with civilian trauma care and guidelines in trauma but lacked adequate volumes of trauma cases. Blended learning supporting health professionals in education and training could be provided through short courses, web-based courses, patient simulations, face-to-face lectures and play an important role. The selection of a particular education and training strategy also hinges on factors such as effectiveness and costs. It is unlikely that any single strategy will satisfy an individual or a unit needs therefore it is essential that a combination of strategies can be used, each offering certain advantages in different settings [1, 3].

#### Quality improvement in education and training

Military medical education and training in the Nordic countries lack methods of transferring outcomes from education into practice. The results have identified a lack of evaluation and feedback of education and training to the military medical education systems, which will undermine the quality of content of courses and programmes as well as practical skills. The lack of standardized methods for evaluating education and training was described as a challenge as was as the lack of feedback from missions to the military medical education programmes. It is a great challenge to provide evidence-based quality and improve education and training for health professionals without feedback on the learning outcomes.

### Views on e-learning and technologies to enhance learning

The results from the study confirm results from the literature overview, showing digital support for learning and teaching is underutilized in the field of military medicine in the Nordic countries. But content quality and attention to the principles of learning are of great importance in e-learning and dramatically affect the learning process for the learner as well as the possibilities of flexible education through the use of blended learning. The educational focus will need to be shifted from the organizational focus to the individual. The military medical education system will need to pay special attention to this possibility, because of the heavy continuing education requirements imposed on health professionals and in relation to provide attractive education and training.

Most of the intreviewees expressed a narrow view of the possibilities of e-learning as support for health professionals in education and training. The interviewees associated e-learning with a technology and especially administrative systems supporting distance studies. Even though the focus of the interviews was on technology for learning, the interviewees centered on individual web-based learning and only with a few exceptions were other kinds of digital technologies such as virtual reality, mobile technologies or more advanced simulators brought up. E-learning was described as the opposite of practical education and training. None of the interviewees thought about using e-learning by combining different educational methods into blended learning, with the purposes of support learning processes, collaboration and release time for health professionals. Despite this, the rapid development of technology also function as a driver and affect the views on how e-learning and blended learning could be used in military medicine in the Nordic countries.

#### Methodological considerations

The study was based on semi-structured interviews with approvement from respective Surgeons General for each country, which also identified the sample. This might have affected the representation of the sample and the outcome of the result but could also be seen as a strength: the Surgeons General enabled access to key military medical units which may otherwise be difficult to reach. Moreover, interviews in all countries were conducted by the same researcher.

## Conclusion

Extended Nordic cooperation on military medical education and training are needed because of the limited volumes of advanced trauma cases. E-learning has the possibility to support learning processes, collaboration, flexibility, and the distribution of education and training, as well as evaluation of content and skill. A key issue to the successful use of e-learning and blended learning is combining educational competence with contextual understanding into a strategy, of how to use digital educational methods.
